# Identification and Characterization of Cell Type–Specific and Ubiquitous Chromatin Regulatory Structures in the Human Genome

**DOI:** 10.1371/journal.pgen.0030136

**Published:** 2007-08-17

**Authors:** Hualin Xi, Hennady P Shulha, Jane M Lin, Teresa R Vales, Yutao Fu, David M Bodine, Ronald D. G McKay, Josh G Chenoweth, Paul J Tesar, Terrence S Furey, Bing Ren, Zhiping Weng, Gregory E Crawford

**Affiliations:** 1 Bioinformatics Program, Boston University, Boston, Massachusetts, United States of America; 2 Biomedical Engineering Department, Boston University, Boston, Massachusetts, United States of America; 3 Institute for Genome Sciences & Policy, Duke University, Durham, North Carolina, United States of America; 4 Hematopoiesis Section, Genetics and Molecular Biology Branch, National Human Genome Research Institute, Bethesda, Maryland, United States of America; 5 National Institute of Neurological Disorders and Stroke, National Institutes of Health, Bethesda, Maryland, United States of America; 6 Ludwig Institute for Cancer Research, University of California San Diego, La Jolla, California, United States of America; Yale University, United States of America

## Abstract

The identification of regulatory elements from different cell types is necessary for understanding the mechanisms controlling cell type–specific and housekeeping gene expression. Mapping DNaseI hypersensitive (HS) sites is an accurate method for identifying the location of functional regulatory elements. We used a high throughput method called DNase-chip to identify 3,904 DNaseI HS sites from six cell types across 1% of the human genome. A significant number (22%) of DNaseI HS sites from each cell type are ubiquitously present among all cell types studied. Surprisingly, nearly all of these ubiquitous DNaseI HS sites correspond to either promoters or insulator elements: 86% of them are located near annotated transcription start sites and 10% are bound by CTCF, a protein with known enhancer-blocking insulator activity. We also identified a large number of DNaseI HS sites that are cell type specific (only present in one cell type); these regions are enriched for enhancer elements and correlate with cell type–specific gene expression as well as cell type–specific histone modifications. Finally, we found that approximately 8% of the genome overlaps a DNaseI HS site in at least one the six cell lines studied, indicating that a significant percentage of the genome is potentially functional.

## Author Summary

There are many different types of gene regulatory elements that control gene expression. Identifying the location of these regulatory elements in the genome, as well as understanding how exactly they control gene expression in different cell types, has been a major challenge. Here, we use a relatively new strategy to identify all gene regulatory elements within a select 1% of the human genome from six diverse human cell types. We find that only 22% of gene regulatory elements are shared among all cell types studied. Among these, 86% are located near annotated transcription start sites and 10% are bound by CTCF, a protein with known enhancer-blocking insulator activity. The gene regulatory elements that are found to be cell type specific are highly correlated with cell type–specific gene expression as well as cell type–specific chromatin modifications. This indicates that we have made a significant step toward understanding why some genes are expressed in all different cell types within the human body, and why others are only expressed in certain cell types.

## Introduction

Biological processes such as proliferation, apoptosis, differentiation, development, and aging require carefully orchestrated spatial and temporal gene expression [1,2]. To understand the molecular mechanisms that underlie global transcriptional regulation, it is essential to identify all the DNA regulatory elements in the human genome. Three methods, DNaseI hypersensitive site (HS) mapping, chromatin immunoprecipitation followed by hybridization to tiled arrays (ChIP-chip), and expression arrays identify gene regulatory elements in different ways. DNaseI HS sites identify regions of open chromatin, which encompass all different types of regulatory elements, including promoters, enhancers, silencers, insulators, and locus control regions (LCR) [3]. However, DNaseI HS mapping does not directly reveal the transcription factor(s) that bind within each DNaseI HS site. ChIP-chip directly identifies the global locations of regulatory factors [4–6], but this method can only be used to study known factors and requires high quality ChIP-grade antibodies. In addition, expression arrays detect genes that are expressed in certain cell types, but do not provide information regarding the factors that cause the cell type–specific expression. Therefore, to completely understand how chromatin structure ultimately regulates gene expression, a multi-pronged integrated experimental approach using all three methods is needed.

We previously used DNase-chip to identify DNaseI HS sites from two cell types across the 1% of the human genome identified by the ENCODE consortium [4]. DNase-chip is a method that works by capturing DNase digested ends, labeling, and hybridizing the material to tiled microarrays. This method is highly sensitive and specific when used to identify valid DNaseI HS sites.

To identify the regulatory elements that control cell type–specific and housekeeping gene expression, we have now performed DNase-chip on the same 1% of the genome from six diverse human cell types: CD4^+^ T cells, GM06990 (B lymphoblastoid), K562 (erythroleukemia), H9 (undifferentiated embryonic stem cell), IMR90 (fetal lung fibroblast), and HeLa S3 (cervical carcinoma). In this study, we find that approximately 22% of all DNaseI HS sites from each cell type are ubiquitously present in all six cell types, while the remainder are a mixture of cell type specific (only present in one cell type) or common (present in two to five cell types). To identify the regulatory roles of these DNaseI HS sites, we performed computational analyses to integrate the DNase data with the ChIP-chip data for two distinct enhancer-binding proteins, one insulator-binding protein, and five histone modifications, as well as expression data from the same six cell lines. The majority (86%) of ubiquitous DNaseI HS sites are within 2 kb of a transcription start site (TSS). Surprisingly, of the remaining ubiquitous HS sites that are distal to TSS, the majority (70%) are bound by CTCF, a factor with known enhancer-blocking activity [7], suggesting that a major role of ubiquitously modified chromatin is to prevent misregulation by local enhancers. In contrast, cell type–specific HS sites are correlated with known enhancer elements [8] and histone-modified regions in a cell type–specific manner. Cell type–specific DNaseI HS sites also contain overrepresented sequence motifs that are biologically relevant and often map near the TSS of genes that exhibit cell type–specific expression. Collectively, these results show that ubiquitous chromatin structures are predominantly associated with promoters and insulators while enhancers tend to associate with cell type–specific chromatin structures.

## Results

### Assessing Data Quality

**Figure 1:**
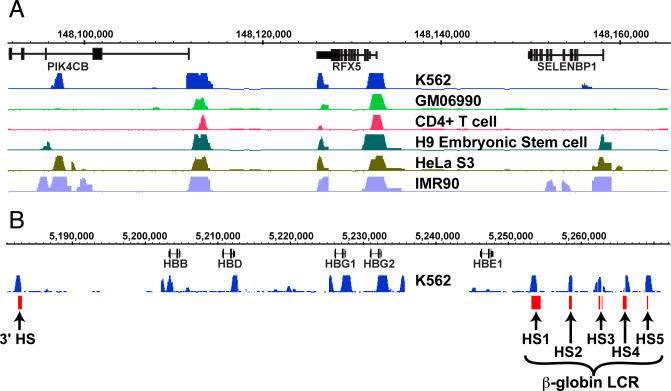
Identification of DNaseI HS Sites from Six Cell Types (A) Representative DNase-chip data from ENCODE region ENr231 (Chr1:148050000–148165000). Note that there are common, ubiquitous, and cell type–specific DNaseI HS sites. (B) DNase-chip from K562 cells identifies all five LCR DNaseI HS sites upstream of the β-globin locus, as well as the 3′ DNaseI HS site (in red). There are additional DNaseI HS sites identified around promoter regions of the globin genes.

For each cell type, DNase-chip data was generated using three concentrations of DNase on each of three biological replicates (See Figure S1 for correlation plots). Averaged data from all replicates (Figure 1A) was used for subsequent analyses, because we have previously shown that averaging data from replicate datasets generates higher sensitivity and specificity [4]. Similar numbers of DNaseI HS sites were identified from each cell type, indicating data consistency (Table 1). To determine specificity for each cell line, we determined the overlap of DNase signal from previously reported “gold standard” negative sets of DNaseI HS sites for CD4^+^ T cells and GM06990 cell lines using real time PCR [4], and calculated >92% specificity for all six cell lines (Table 1). As a second measure of specificity, we determined the numbers of significant signals that are detected in two ENCODE regions (ENr112 and ENr313) that are depleted for TSS, DNaseI HS sites, active histone modifications, and ChIP-chip signals. Significant signals that map within these two regions are considered likely false positives. For each cell line, only a few significant signals were observed in these two regions, which also indicates high specificity (Table 1). We have previously shown that sensitivity of DNase-chip experiments from CD4^+^ T cells and GM06990 was >86% [4]. To assess the sensitivity of these additional cell lines, we examined five well-characterized DNaseI HS sites that make up the globin locus control region [9,10]. We robustly detect all five DNaseI HS LCR sites in K562 cells, as well as the well-characterized 3′ DNaseI HS site [11] (Figure 1B). In addition, results from all six cell lines show a significant enrichment for TSS and CpG islands, one of the hallmarks of active chromatin (Table 1 and unpublished data). Together, these results indicate that the sensitivity and specificity in the four newly studied cell lines are consistent with those in CD4^+^ T and GM06990 cells. All DNase-chip data described here is publicly available on the University of California Santa Cruz (UCSC) genome browser [12] (http://genome.ucsc.edu).

**Table 1:**
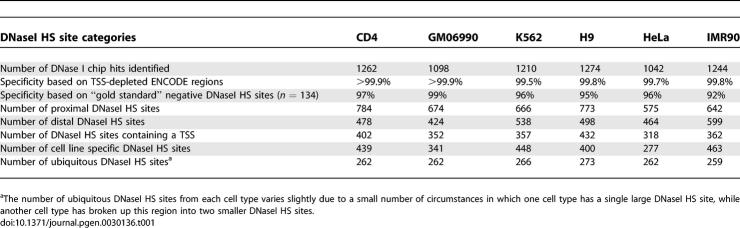
DNase-Chip Statistics for Different Cell Lines

### Properties of DNaseI HS Sites

#### Classification by cell type.

**Figure 2:**
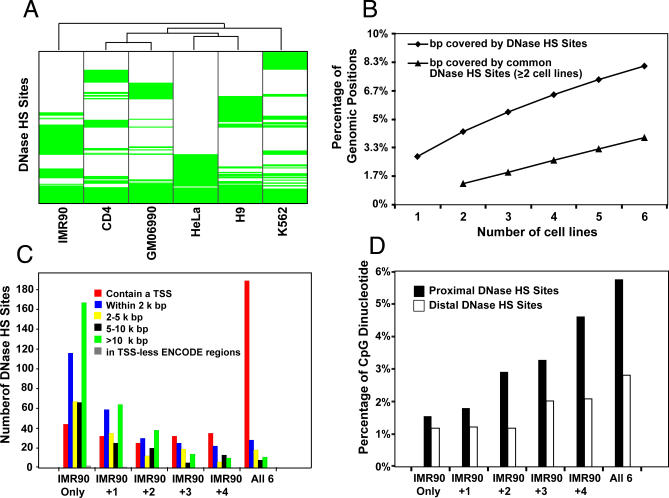
Identification and Characterization of DNaseI HS Sites (A) Clustering of six cell lines based on DNaseI HS site profiles. ENCODE regions were divided into 2-kb blocks and a binary DNaseI HS site profile was calculated for each cell line (1 for blocks containing a DNaseI HS sites hit, displayed in green, and 0 otherwise, uncolored). Cell lines were clustered based on their DNaseI HS sites hit profiles using Wards hierarchical clustering [41] with Euclidian distance as the metric. Ubiquitous DNase sites are grouped at the bottom. (B) Cumulative percentage of the genome covered by DNaseI HS sites from increasing number of cell lines. Diamonds represent cumulative percentage of the genome covered by DNaseI HS sites from any cell line. Triangles represent cumulative percentage of the genome overlapped by DNaseI HS sites shared by at least two cell types. Each point is an averaged value of all possible cell line combinations. (C) Location of DNaseI HS sites relative to TSS. DNaseI HS sites from IMR90 cells were first categorized as unique to IMR90, common with other cell types, or ubiquitous in all six cell types. Data centering on other cell types are identical (unpublished data). Distances of each DNaseI HS site were calculated to the nearest TSS. (D) CpG dinucleotide distribution. The percentage of CG dinucleotide was determined for proximal and distal DNaseI HS sites that were unique to IMR90, common with additional cell types, or ubiquitous within all six cell types.

DNaseI HS sites are classified as cell type specific (only found in one out of six cell lines), common (found in two to five cell lines), or ubiquitous (found in all six cell lines) (Table 1; Figure 2A). Between any two cell lines, fewer than 50% of DNaseI HS sites overlap. The highest overlapping datasets were from the two lymphocyte cell lines, CD4^+^ and GM06690. On average for each cell type, 32% of DNaseI HS sites are cell type specific, 46% are common, and 22% are ubiquitous. A total of 3,904 distinct DNaseI HS sites were identified from the six cell types.

#### DNaseI HS sites and gene expression profiles show similar lineage specificity.

To test whether we can determine cell type specificity from DNaseI HS sites, we compared cluster dendrograms from both DNaseI HS sites and expression data. Both dendrograms have the closest clustering occurring between CD4^+^ T cell and GM06990 B lymphoblastoid (Figures 2A and S2). This is to be expected, as these two cell types are derived from a common lymphoid progenitor. Interestingly, K562, which is an erythroleukemia cell line, does not cluster closely with CD4^+^ T and GM06990 using either DNase or expression data. However, other studies have shown that K562 cells have characteristics distinct from B and T cells [13].

#### Determining DNaseI HS site saturation.

To determine whether we have identified most DNaseI HS sites in the ENCODE regions, we computed the cumulative percentage of base pairs as a function of the number of cell lines tested. As additional cell lines are included, the total percentage of base pairs of the ENCODE regions covered by DNaseI HS sites increases steadily, reaching ∼8% at six cell lines (Figure 2B). We wanted to know whether the new sites are predominantly those that are unique to one cell type. DNaseI HS sites that are observed in only one cell type tend to be less sensitive to DNaseI cleavage and hence may be more affected by microarray noise than sites observed in multiple cell types. Nonetheless, as discussed in the last section of Results, cell type–specific DNaseI HS sites are enriched in the regions bound by enhancer proteins and regions with modified histones, thus, they contain bone fide regulatory elements. We do not detect a significant leveling off after the addition of the sixth cell type, even if we only analyze DNaseI HS sites that are common in at least two cell types. This indicates that additional cell lines must be tested in the future to identify most DNaseI HS sites.

#### Location of DNaseI HS sites relative to genes.

We calculated the distance of DNaseI HS sites to the nearest TSS, with the TSS set defined in a comprehensive way by the Integrated Analysis group of the ENCODE consortium [14] (Figure 2C). Thirty-four percent of cell type–specific DNaseI HS sites are proximal to a TSS (<2 kb). In stark contrast, 86% of ubiquitous DNaseI HS sites are proximal to TSS. The distribution of proximal DNaseI HS sites in terms of the numbers of tissues in which they occur is significantly different from those of other DNaseI HS site categories in Figure 2C (*p*-value < 2.2 × 10^−16^ by Wilcoxon test). The dramatic increase in proximal DNaseI HS sites in six cell lines over five cell lines indicates the high quality of the DNase data, because one would expect a more gradual shift for data with low sensitivity and specificity. While proximal DNaseI HS sites are overrepresented in the genome, distal DNaseI HS sites are underrepresented and become increasingly underrepresented the further away from the TSS (Figure S3). Therefore, distal DNaseI HS sites are not uniformly distributed in the genome but instead are located closer to genes.

#### CpG dinucleotide distribution.

Because CpG islands are generally associated with housekeeping promoters of mammalian genes, we asked whether the percentage of CpG dinucleotides differs among unique, common, and ubiquitous DNaseI HS sites. Cell type–specific DNaseI HS sites tend to have similar percentages of CG dinucleotides regardless of whether the DNaseI HS sites are proximal (<2 kb) or distal (>2 kb) to a TSS (Figure 2D). DNaseI HS sites that are common to more cell types are more CpG rich than cell type–specific sites, and the slope of this increase is much greater for proximal sites than for distal sites (Figure 2D). A similar but more moderate trend is detected for G + C mononucleotide (Figure S4).

### Ubiquitous Proximal DNaseI HS Sites

**Figure 3:**
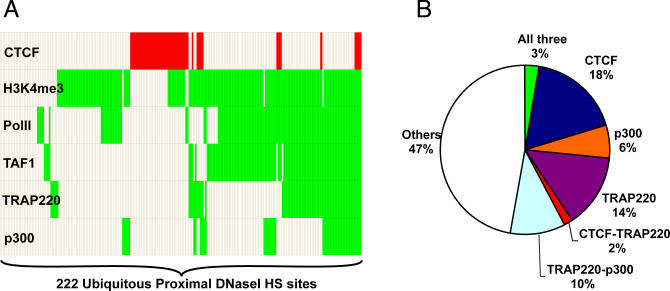
Ubiquitous Proximal DNaseI HS Sites Overlap with Known Factors (A) Chart identifying the different factors that overlap 222 ubiquitous DNaseI HS sites that map to TSS, including RNA Pol II, TAF1, TRAP220, p300, CTCF, and the H3K4me3 histone mark. Red indicates overlap with CTCF, green indicates overlap with other marks, and unfilled boxes represent no overlap. (B) Pie chart representing the percentage of ubiquitous DNaseI HS sites that overlap with various factors (histone modifications not shown for clarity purposes).

For the 222 proximal DNaseI HS sites (<2 kb from TSS) that are ubiquitous in all six cell lines, 78% overlap recently published ChIP-chip data specific to basal promoter factors (RNA PolII and TAF1) [15], enhancers (p300 and TRAP220) [8], or the insulator factor CTCF [16] (Figure 3A and 3B). The majority (81%) of DNaseI HS sites bound by p300 or TRAP220 also bind Pol II or TAF1. However, only 22% DNaseI HS sites bound by CTCF are also bound by TAF1 or Pol II, suggesting that CTCF binding to the promoter decreases the likelihood of binding by other factors examined. For the ubiquitous proximal DNaseI HS sites that do not overlap known promoter factors, 53% overlap with the ChIP hits of H3K4me3, a histone modification mark for active promoters (Figure 3A) [17].

### Ubiquitous Distal DNaseI HS Sites

#### The majority of distal ubiquitous DNaseI HS sites contain insulators.

**Figure 4:**
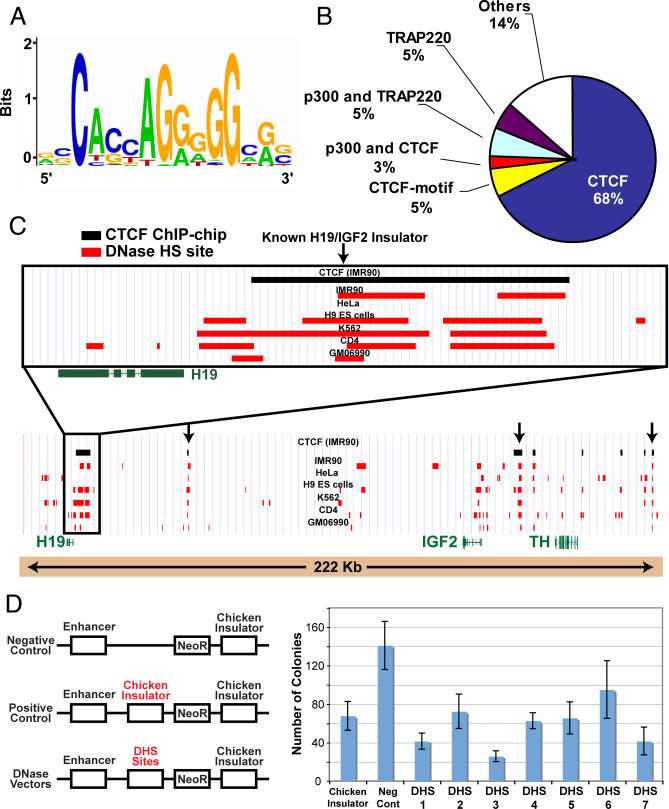
The CTCF Motif Is Identified in Ubiquitous Distal DNaseI HS Sites (A) Motif identified using de novo motif-finding algorithm using the minimal intersecting regions of ubiquitous distal DNaseI HS sites. The overall height of the stack of letters in a position indicates the sequence conservation at that position, while the height of letters within the stack indicates the relative frequency of each base at that position**.** (B) Percentage of ubiquitous distal DNaseI HS sites that overlap with CTCF ChIP-chip data, CTCF motif, or other enhancer elements. (C) Example of clustered ubiquitous DNaseI HS sites that overlap CTCF in the *H19/IGF2* locus. Arrows indicate ubiquitous DNaseI HS sites overlapping with CTCF. (D) Cell culture insulator assays demonstrate that DNaseI HS sites (that overlap CTCF) display enhancer-blocking activity. Higher colony counts in G418 media indicate no enhancer-blocking activity, while lower colony counts indicate positive enhancer-blocking activity. A previously described chicken insulator was used as a positive control [11].

Of the 259 DNaseI HS sites that are ubiquitous in all six cell lines, 37 are distal (>2 kb) to a TSS. To identify the protein(s) that putatively bind to these regions, the minimal intersecting region for each ubiquitous site was analyzed by the de novo motif-finding algorithm MEME [18]. The most significant motif (*p*-value < 2.2 × 10^−16^; Figure 4A) was not in the TRANSFAC [19] or Jaspar [20] databases, but is nearly identical to the motif recently discovered in a genome-wide ChIP-chip study with an antibody against CTCF in IMR90 cells [16]. Using this ChIP-chip data, we find that 70% (26/37) of the ubiquitous distal DNaseI HS sites overlap with CTCF binding sites (Figure 4B; Tables S1 and S2). An additional four ubiquitous distal DNaseI HS sites that do not overlap with CTCF hits contain the CTCF motif. Some of these 26 distal ubiquitous sites that overlap CTCF are clustered in the genome. For example, three DNaseI HS sites are in the *IGF2/H19* locus (ENCODE region ENm011), clearly isolating the *H19*, *IGF2*, and *TH* genes (Figure 4C). The well-characterized imprinting insulator between *H19* and *IGF2* [21] is not one of these three; however, it overlaps with a DNaseI HS site that is present in five cell lines. In addition, four ubiquitous DNaseI HS sites overlap CTCF hits in the *HoxA* locus (Figure S5).

#### The CTCF motif is predictive of CTCF binding.

The CTCF motif can be found in 88 (55%) of the 160 DNaseI HS sites that overlap with CTCF hits (*p*-value cutoff of 10^−5^ as computed by the MAST algorithm [22]; Table S2). Most (85%) of these 88 DNaseI HS sites contained only a single CTCF motif site. DNaseI HS sites that contain two (*n* = 23) or three motifs (*n* = 4) were not more enriched for CTCF ChIP-chip hits (unpublished data), indicating a single CTCF motif is sufficient to facilitate significant CTCF binding. CTCF motif sites in both distal and proximal DNaseI HS sites are significantly more conserved than neighboring genomic regions based on phastCons [23] conservation scores (Figure S6). Approximately 19% of DNaseI HS sites in IMR90 that do not overlap CTCF ChIP-chip data (139/1084) contain the CTCF motif (Table S2). Although these CTCF motif sites are on average less conserved than those that overlap CTCF ChIP-chip hits (Figure S6), the subset in distal DNaseI HS sites are still significantly more conserved than neighboring genomic regions (leftmost bar in Figure S6), indicating that they may bind CTCF in living cells.

#### DNaseI HS sites contain functional enhancer-blocking elements.

We performed cell culture enhancer-blocking assays [7] on seven CTCF motif-containing DNaseI HS sites; six of these are ubiquitous and the other one is common in five cell types (Table S3). All seven clones display significant enhancer-blocking activity (Figure 4D; *p*-value = 0.002), including the DNaseI HS site that does not overlap a CTCF ChIP-chip hit (DHS4). Three of the DNaseI HS sites are proximal to TSS (DHS1, DHS4, and DHS6). Although we only tested a small number of DNaseI HS sites, our results indicate that DNaseI HS sites that occur in many cell types and contain the CTCF motif are likely functional insulators. In addition, proximal DNaseI HS sites near TSS can also function as insulators.

#### CTCF is preferentially bound to ubiquitous and common DNaseI HS sites.

**Figure 5:**
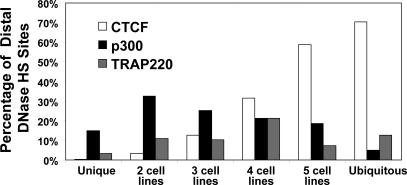
Percentage of Distal Unique, Common, or Ubiquitous DNaseI HS Sites That Overlap with CTCF, p300, and TRAP220 CTCF binding makes up a greater percentage of more common and ubiquitous distal DNaseI HS sites, while p300 and TRAP220 binding makes up a greater percentage of cell type–specific and less-common DNaseI HS sites.

Of the 225 CTCF ChIP-chip hits that map within ENCODE regions, 160 (71%) overlap with DNaseI HS sites identified in IMR90 cells. The percentage of DNaseI HS sites that overlap CTCF ChIP-chip hits steadily increases for DNaseI HS sites that are more common, with the highest percentage occurring within ubiquitous DNaseI HS sites (Figure 5). This is in contrast to the binding sites for p300 and TRAP220, proteins with enhancer activity, which are preferentially detected in cell type–specific and less common DNaseI HS sites (Figure 5). This indicates that insulators, but not enhancers, comprise the majority of ubiquitous distal regulatory elements.

### Cell Type–Specific and Common DNaseI HS Sites

#### DNaseI HS sites colocalize with histone-modified regions in a cell line–specific manner.

**Figure 6:**
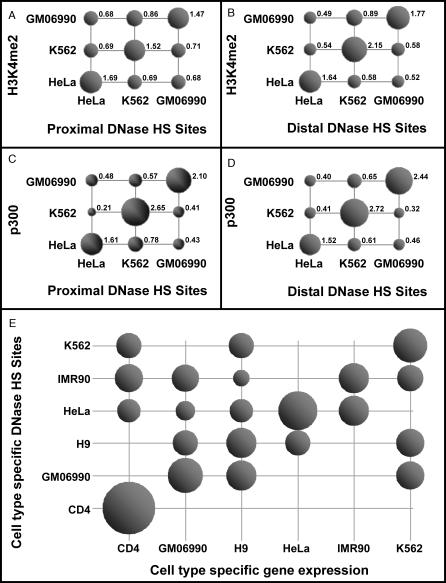
DNaseI HS Sites Colocalize with Histone Modifications, p300 Binding, and Gene Expression in a Cell Type–Specific Manner The enrichment factors of proximal (A) or distal (B) DNaseI HS sites with H3K4me2 ChIP-chip hits from three different cell types. The enrichment factors of proximal (C) or distal (D) DNaseI HS sites with p300 ChIP-chip hits. (E) Cell type–specific DNaseI HS sites (*y*-axis) are mapped relative to transcription start sites of genes with cell type–specific expression (*x*-axis). Size of bubbles represents the enrichment. When no bubble is present, the value is zero (complete depletion).

Previously, ChIP-chip for five histone modifications (H3K4me2, H3K4me3, H3ac, and H4ac) was performed on three cell lines (HeLa, GM06990, and K562) and ChIP-chip for H3K4me1 was performed on two cell lines (HeLa and GM06990) [17]. We calculated the number of DNaseI HS sites that overlap ChIP-chip hits for each histone modification in 3-by-3 cell line combinations (2-by-2 in the case of H3K4me1). Ubiquitous DNaseI HS sites often overlap with ubiquitous histone modification hits, in particular with H3K4me3 and H3ac, which are strong markers for the 5′ ends of active genes. This is consistent with our aforementioned results indicating that 86% ubiquitous DNase HS sites are promoters. Respectively, there are 78 and 103 ubiquitous H3K4me3 and H3ac hits in the ENCODE regions; 59 and 80 of them overlap with ubiquitous DNaseI HS sites, respectively. Ubiquitous DNaseI HS sites and ubiquitous histone modification hits were excluded from the remaining analysis in this section, because they merely increase all counts of overlap. The counts were divided by the corresponding row sum and column sum and multiplied by the matrix sum to obtain enrichment values, which is done in the same way as the χ^2^ test (see Figure S7 for detailed explanation). In Figure 6A and 6B, we plot the enrichment factor for H3K4me2 in a 3-by-3 grid (see Figure S8 for other histone modifications). The diagonal matched cell line enrichment values (all >1) are much larger than off-diagonal mismatched cell line values (<1 for all comparisons except H3ac in the K562-HeLa comparison), indicating that DNaseI HS and ChIP-chip experiments are both detecting similar genomic regions that reflect cell type specificity. This agreement is particularly striking given that the DNaseI HS and ChIP-chip experiments were performed in different labs and on different microarray platforms [17] (the histone modification experiments were on spotted PCR arrays).

#### DNaseI HS sites colocalize with p300-binding regions in a cell line–specific manner.

We performed overlap analysis on DNaseI HS sites and p300 ChIP-chip hits in three cell types (HeLa, K562, and GM06990), in the same way as described above for histone modifications. Again, ubiquitous DNaseI HS sites and ubiquitous p300 hits were excluded from this analysis. The results are shown in Figure 6C and 6D, for proximal and distal DNaseI HS sites separately, both indicating strong colocalization.

#### Cell type–specific DNaseI HS sites colocalize with cell type–specific gene expression.

We hypothesized that cell type–specific DNaseI HS sites are involved in cell type–specific gene regulation, and therefore expected them to colocalize with (within 2 kb of) the TSS of genes active in the corresponding cell line. Because only a few genes were strictly expressed exclusively in one cell line within the ENCODE regions, the definition of cell type–specific genes was relaxed to include genes expressed in no more than two cell lines in addition to the cell line of interest. The 6-by-6 enrichment matrix (Figure 6E) was constructed in the same way as described above for histone modifications. The diagonal enrichment values (matched cell lines) are larger than off-diagonal values (mismatched cell lines), indicating that cell type–specific DNaseI HS sites tend to colocalize with genes that are expressed in the corresponding cell types (*p*-value = 1.15 × 10^−4^ by Wilcoxon ranked sum test). The significance is maintained if we loosen the proximity criteria to DNaseI HS sites that are within 5 kb or 10 kb of a cell type–specific TSS (unpublished data).

#### Cell type–specific DNaseI HS sites are enriched for biologically relevant motifs.

To identify putative regulatory factors that bind cell type–specific DNaseI HS sites, we analyzed these sites with Clover [24], a motif-finding algorithm that identifies motifs from the TRANSFAC database that are enriched in a set of sequences, namely the DNaseI HS sites specific to a cell type. We used two sets of background sequences for computing the enrichment: the union set of all ChIP-chip hits generated by the ENCODE Transcription Regulation group at the 5% false discovery rate cutoff [14], and random dinucleotide shuffling of the input sequence set (DNaseI HS sites specific to a cell line). We obtained similar results from both sets of background sequences. Motifs enriched in each cell line were identified for DNaseI HS sites proximal or distal relative to the TSS (Table 2). Many of the overrepresented motifs are functionally relevant to the cell type from which the DNase data was generated. For example, the TAL1 motif [25,26], enriched in CD4^+^ T specific DNaseI HS sites, binds a well-known transcription activator involved in hematopoietic stem cell function and the development of T cell acute lymphoblastic leukemia [27]. K562 DNaseI HS sites are enriched for the GATA1 motif, which is a factor known to be involved in erythroid maturation [28]. H9 ES cell DNaseI HS sites are enriched for the Octamer [29], Sox, and STAT family motifs, which have been reported to be involved in pluripotency and early differentiation [30,31]. The AP-1 motif [32] is enriched in HeLa DNaseI HS sites [33]. AP-1 is especially enriched in those HeLa DNaseI HS sites that overlap with p300 ChIP-chip hits (*p*-value < 2.2 × 10^−16^ by χ^2^ test; Table S4). The AP-1 components, c-jun and c-fos, are among the many proteins known to interact with p300 [34]. Because the AP-1 motif is the most enriched motif in p300 ChIP-chip hits (unpublished data), this suggests that AP-1 contributes to the DNA binding specificity of p300.

**Table 2:**
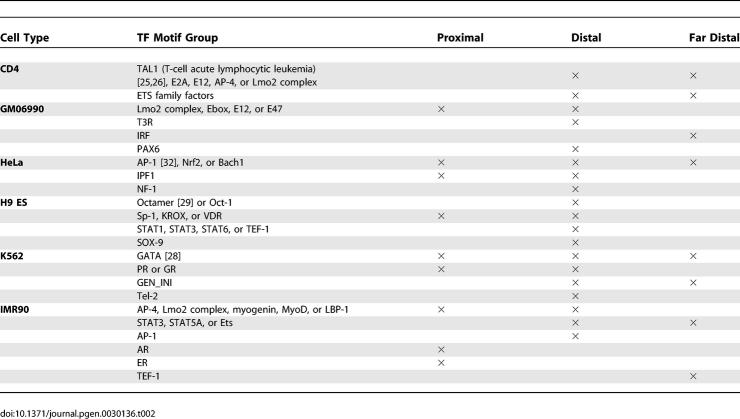
Tissue-Specific DNaseI HS Sites Are Enriched in Motifs Indicative of the Corresponding Cell Types

## Discussion

We present DNase-chip data from six cell lines and classify the DNaseI HS sites into cell type–specific, common (found in more than one but not all cell lines), and ubiquitous categories. Only 22% of all DNaseI HS sites are ubiquitous in all cell lines, indicating that the majority of gene regulatory elements are involved in cell type–specific function.

The identification of ubiquitous DNaseI HS sites provides clues to the function of housekeeping chromatin structures that are maintained in most cell types. We detected 259 such ubiquitous sites in the ENCODE regions. Approximately 86% of ubiquitous DNaseI HS sites are proximal to TSS and map to basal transcription factor binding sites, indicating that these regions function as housekeeping promoters. The majority of ubiquitous distal DNaseI HS sites bind to CTCF, a protein with known enhancer-blocking insulator activity [7], indicating that CTCF is involved in stable chromatin structure and gene expression maintenance across many cell types. Because most ubiquitous sites bind to either basal transcription machinery or CTCF, we conclude that ubiquitous DNaseI HS sites function primarily as promoters and insulators, but not enhancers. Cell type–specific DNase HS sites, however, are more enriched for protein binding sites with known enhancer activity, cell type–specific histone modifications, and cell type–specific gene expression.

Although our DNaseI HS site data was limited to 1% of the human genome, the integration over multiple data types allowed us to conclude that we have uncovered many different types of functional regulatory elements. In the future, as additional cell types are analyzed using whole genome DNase-chip, we will be able to better characterize DNaseI HS sites that are truly cell type specific, as well as those that are shared between cell types of similar lineages. We expect that genome-wide analysis from additional cell types, under different cellular conditions, or at different developmental stages, will provide for more powerful de novo motif discovery, similar to our identification of CTCF, for identifying and characterizing unknown factors that regulate temporal and spatial gene expression.

Our integrated approach combines the strengths of four high-throughput technologies (DNase-chip, ChIP-chip, expression array, and motif discovery). DNase-chip can identify all types of regulatory elements in a single experiment and integration with other datasets has allowed us to delineate the functions of subsets of DNaseI HS sites. This approach will be increasingly more powerful as more high-throughput datasets become available and will be an important part of ensuring that no regulatory element is missed. Nonetheless, our analysis is missing an important component—we cannot identify the target gene(s) of a DNaseI HS site. Technologies such as chromosome conformation capture carbon copy (5C) [35] are ideal for detecting large numbers of long-range interactions between genomic elements. Since 5C works best by anchoring to known regulatory elements, DNaseI HS sites identified in our study can be used to significantly reduce the search space.

DNaseI HS sites can be used as a general tool for evaluating future ChIP-chip datasets that have been performed on only one of the cell types described here, to determine whether those factors bind genomic DNA in a cell type–specific or ubiquitous manner. This is illustrated for the p300 ChIP-chip data in Figure S9, which shows that the percentages of p300 binding (performed in HeLa cells) are highest for HeLa-specific distal DNaseI HS sites. Other examples of cell line-specific marks are H3K4me1 and H3K4me2 (Figure S9). In contrast, H3K4me3, H3ac, H4ac, and CTCF show less cell line specificity (Figure S10). Our DNaseI HS data can also be used to help identify unknown transcription factors binding and unknown histone modification patterns. For example, only 60% of proximal DNaseI HS sites overlap with the five histone modifications we examined in this study. Future studies will be needed to identify the histone modification(s) that are associated with these regions.

While DNaseI HS sites from each cell type cover approximately only 2%–3% of the genome, the combined DNase data from six cell types covers roughly 8% of the genome. Since the actual functional regulatory sequences (i.e., protein binding sites) may make up a fraction of each DNase HS site, the actual percentage of functional DNA may be smaller. As we have not detected a significant decrease in the number of new DNaseI HS sites identified with the addition of each cell type, this indicates that a large percentage of the genome may be functional in all possible cell types, disease states, and responses to external stimuli. Whole genome identification of all DNaseI HS sites using DNase-chip [4] or DNase-sequencing [36] methods will play a key role in identifying and ultimately understanding the function of all functional noncoding DNA sequences.

## Materials and Methods

### Identification of DNaseI hypersensitive sites.

DNase-chip was performed as previously described [4]. Briefly, intact nuclei were digested with optimized amounts of DNase. DNase digested ends were blunted, ligated to biotinylated linkers, sonicated, and enriched on a streptavidin column. Sheared ends were blunted and ligated to nonbiotinylated linkers. DNase-enriched material was amplified by linker-mediated PCR, labeled, and hybridized to NimbleGen ENCODE arrays. Randomly sheared DNA was used as a reference control. For each cell type, DNase-chip material was generated from three biological replicates and three different DNase concentrations (total of nine hybridizations per cell type). Raw ratio data from each cell type was averaged and significant signals were identified using ACME (*p*-value = 0.001). All DNase-chip data is publicly available on the UCSC genome browser [12] (http://genome.ucsc.edu).

### Embryonic stem cell culture.

Human ES cell line H9 [37] (WiCell Research Institute; National Institutes of Health Code WA09) was cultured on a feeder layer of mitotically inactivated mouse embryo fibroblasts in medium consisting of DMEM-F12 supplemented with 20% KSR (Invitrogen, http://www.invitrogen.com), 5 ng/ml FGF2 (R&D Systems, http://www.rndsystems.com/), 2mM L-glutamine, 0.1mM 2-mercaptoethanol, and 1× nonessential amino acids. For analysis, hES cell colonies were separated away from the feeder layer and processed for DNaseI hypersensitive site mapping. The undifferentiated state of the cultures was determined by morphology, immunohistochemistry, and Affymetrix expression array (http://www.affymetrix.com) analysis.

### Expression arrays.

Total RNA was extracted from CD4^+^ T cells, GM06990, HeLa S3, K562, and H9 undifferentiated stem cells using Trizol (Invitrogen). RNA was analyzed by Bioanlyzer to confirm high-quality 18s and 28s ribosomal bands (Agilent, http://www.home.agilent.com/), labeled, and hybridized to Affymetrix U133 Plus 2.0 arrays. The expression data for IMR90 was publicly available (http://licr-renlab.ucsd.edu/download.html). All data was normalized together using RMA [38] through the BioConductor project's Affymetrix package (http://www.bioconductor.org). Only genes expressed in the ENCODE regions were used for the analysis. Gene expression was categorized as expressed or not expressed by the Affymetrix A/P call.

### Enhancer block cell culture assays.

The enhancer blocking assay was performed as described previously [7]. Briefly, the β-globin DNaseI HS2 site, which is a known enhancer element [39], was cloned upstream of a NeoR gene. Putative insulators were cloned between the enhancer and NeoR gene. The previously described chicken insulator was used a positive control [40]. DNaseI HS sites proximal or distal to TSS that also overlapped CTCF binding sites were cloned into the enhancer block vector. All plasmids were purified from three independent bacterial cultures, linearized, and each DNA prep was electroporated independently into K562 cells. Each electroporation was plated in triplicate (total of nine experiments per plasmid). The next day, cells were transferred to soft agar media containing G418. After 16 days, plates were scanned and colonies were counted.

### ChIP and TSS data analysis.

Publicly available ChIP-chip data of CTCF, p300, RNA Pol II, TRAP220 was obtained from http://licr-renlab.ucsd.edu/download.html. ChIP-chip data for five histone modifications were obtained from the UCSC genome browser (http://genome.ucsc.edu). The coordinates for TSS were obtained from the ENCODE pilot study [14] (TSS set ABCDE defined in supplement 3.5 therein). These data were mapped onto the DNaseI HS sites in each cell line based on their overlapping coordinates. To determine whether distal DNaseI HS sites are still statistically near genes, we binned all DNaseI HS sites according to their distances to the closest TSS. We computed an enrichment score for each bin, defined as the ratio between the number of DNaseI HS sites and the number of all possible positions in the ENCODE regions in the same distance bin.

### Motif identification.

MEME [18] with the “-zoop” option was used to identify the CTCF sites in the ubiquitous distal DNaseI HS sites. Clover [24] was used to identify motifs overrepresented in cell type–specific DNaseI HS sites at each distance category: proximal (<2 kb), distal (between 2 kb and 10 kb) and far distal (>10 kb). Two background sets were used (union of ChIP-chip hits and random dinucleotide shuffling of input sequences). Overrepresented motifs (*p-*values < 0.01) from the TRANSFAC database were reported. Overlapping motifs were reported as groups.

## Supporting Information

Figure S1 Correlation Plots of DNase-Chip Data from Each of Three Biological Replicates and Three Different DNase Concentrations (Two Replicates and Four DNase Concentrations for H9) Each correlation plot corresponds to raw ratio values from each DNase-chip replicate (*x*-axis) compared to the averaged raw ratio values of all DNase-chip replicates from each cell type (*y*-axis). The Pearson correlation coefficients (R) are shown at the bottom of each plot. Correlation coefficients are lower for CD4^+^ T cells and the GM06990 cell lines, but this data was previously shown by extensive quantitative PCR validation [4] to have high sensitivity (88%) and specificity (97%).
(619 KB TIF)

Figure S2 Clustering Dendrograms from Six Cell Types Using DNaseI HS sites Is Similar to Clustering Using Gene Expression Data Six cell lines were clustered using DNaseI HS sites data in the same way as described in Figure 2A. For expression data, the Euclidian distances are calculated from the Robust Multichip Average (RMA) [38] normalized expression levels between each cell line for genes that map within ENCODE regions. Other algorithms also cluster CD4^+^ and GM06990 first, a sign that this grouping is robust and biological (unpublished data). To test whether this was a result of analyzing genes within 1% of the genome, we performed the same clustering using genome-wide expression data and find that CD4^+^ T and GM06990 cells remain the closest-clustering cell lines, while K562 remains more distantly clustered (unpublished data).
(635 KB TIF)

Figure S3 DNaseI HS Sites Are Enriched for Gene-Rich Regions of the Genome For each cell line, DNaseI HS sites were assigned to distance bins based on the distances to the nearest transcription start site (TSS). Similarly, each genomic position in the ENCODE regions is assigned to a bin based on its distance to the nearest TSS. To calculate the enrichment of DNase sites, the percentage of DNaseI HS sites in each distance bin is normalized by the percentage of positions in each distance bin. DNaseI HS sites near TSS were enriched and DNaseI HS sites were less likely to be found at large distances from a TSS.
(279 KB TIF)

Figure S4 GC Content for Proximal and Distal DNaseI HS Sites That Are Unique to IMR90 Cells, Common to IMR90 Plus Additional Cell Types, and Ubiquitous to All Six Cell Types (272 KB TIF)

Figure S5 Ubiquitous DNaseI HS Sites within the *HoxA* Locus Overlap with CTCF Binding Arrows represent ubiquitous DNaseI HS sites.
(776 KB TIF)

Figure S6 Distribution of PhastCons Scores for CTCF Motifs of Different Categories The *y*-axis is the percentage of base pairs with phastCons scores of different ranges. PhastCons takes a multiple sequence alignment, a phylogenetic model for conserved regions, and a phylogenetic model for nonconserved regions as input. It scans along for regions that better fit the conserved model than the nonconserved model and output the probability that each base is in such a region as the conservation score for that base. The categories are: “Distal non-overlap,” CTCF motifs located in distal DNaseI HS sites (greater than 2 kb from any TSS) that do not overlap with CTCF ChIP chip hit regions; “Distal overlap,” CTCF motifs located in distal DNaseI HS sites that overlap with CTCF hit regions; “Proximal non-overlap,” CTCF motifs located in proximal DNaseI HS sites (less than 2 kb from a TSS) that do not overlap with CTCF hit regions; and “Proximal overlap,” CTCF motifs located in proximal DNaseI HS sites that overlap with CTCF hit regions. For each category, the genomic regions 100 bp downstream from the motif coordinates were used as the control.
(1.0 MB TIF)

Figure S7 Step-by-Step Illustration for Computing the Enrichment of the Overlap between Common or Cell Type–Specific Distal DNase HS Sites and Common or Cell Type–Specific ChIP-Chip Hits Shown here is an example using H3K4me2 data (the final matrix is plotted in Figure 6B).
(667 KB TIF)

Figure S8 Cell Type–Specific DNaseI HS Sites Colocalize with Cell Type–Specific Histone Modifications (H3K4me1, H3K4me3, H3ac, and H4ac) Each plot shows the enrichment of the overlap between proximal or distal DNaseI HS sites and each histone modification hit from three different cell types. See main text for the enrichment of H3K4me2 and the description of the enrichment calculation.
(3.5 MB TIF)

Figure S9 Transcription Factors and Histone Modifications That Display Strong Cell Type–Specific Binding ChIP-chip from HeLa cells are compared to proximal and distal DNaseI HS sites that are unique to HeLa cells, common, or ubiquitous to all six cell types.
(2.7 MB TIF)

Figure S10 Transcription Factors and Histone Modifications That Display Weak Cell Type–Specific Binding ChIP-chip from HeLa and IMR90 cells are compared to proximal and distal DNaseI HS sites that are unique to HeLa or IMR90 cells, common, or ubiquitous to all six cell types.
(3.2 MB TIF)

Table S1 Click here for additional data file. The 26 Ubiquitous Distal DNaseI HS That Overlap CTCF ChIP-Chip Hits
(77 KB DOC)

Table S2 Click here for additional data file. Contingency Tables of DNaseI HS versus CTCF Hits
(29 KB DOC)

Table S3 Click here for additional data file. DNaseI HS Sites Used in Enhancer-Blocking Assay (hg17 Coordinates)
(39 KB DOC)

Table S4 Click here for additional data file. Enrichment of AP-1 Motif (TGASTCA) in DNaseI HS Sites That Overlap p300 Hits
(27 KB DOC)

## Abbreviations

HS: hypersensitive
LCR: locus control region
TSS: transcription start site

## References

[pgen-0030136-b001] Lemon B, Tjian R (2000). Orchestrated response: A symphony of transcription factors for gene control. Genes Dev.

[pgen-0030136-b002] Orphanides G, Reinberg D (2002). A unified theory of gene expression. Cell.

[pgen-0030136-b003] Gross DS, Garrard WT (1988). Nuclease hypersensitive sites in chromatin. Annu Rev Biochem.

[pgen-0030136-b004] Crawford GE, Davis S, Scacheri PC, Renaud G, Halawi MJ (2006). DNase-chip: A high-resolution method to identify DNase I hypersensitive sites using tiled microarrays. Nat Methods.

[pgen-0030136-b005] Orlando V (2000). Mapping chromosomal proteins in vivo by formaldehyde-crosslinked-chromatin immunoprecipitation. Trends Biochem Sci.

[pgen-0030136-b006] Ren B, Robert F, Wyrick JJ, Aparicio O, Jennings EG (2000). Genome-wide location and function of DNA binding proteins. Science.

[pgen-0030136-b007] Bell AC, West AG, Felsenfeld G (1999). The protein CTCF is required for the enhancer blocking activity of vertebrate insulators. Cell.

[pgen-0030136-b008] Heintzman ND, Stuart RK, Hon G, Fu Y, Ching CW (2007). Distinct and predictive chromatin signatures of transcriptional promoters and enhancers in the human genome. Nat Genet.

[pgen-0030136-b009] Forrester WC, Takegawa S, Papayannopoulou T, Stamatoyannopoulos G, Groudine M (1987). Evidence for a locus activation region: The formation of developmentally stable hypersensitive sites in globin-expressing hybrids. Nucleic Acids Res.

[pgen-0030136-b010] Tuan D, London IM (1984). Mapping of DNase I-hypersensitive sites in the upstream DNA of human embryonic epsilon-globin gene in K562 leukemia cells. Proc Natl Acad Sci U S A.

[pgen-0030136-b011] Saitoh N, Bell AC, Recillas-Targa F, West AG, Simpson M (2000). Structural and functional conservation at the boundaries of the chicken beta-globin domain. EMBO J.

[pgen-0030136-b012] Kent WJ, Sugnet CW, Furey TS, Roskin KM, Pringle TH (2002). The human genome browser at UCSC. Genome Res.

[pgen-0030136-b013] Klein E, Ben-Bassat H, Neumann H, Ralph P, Zeuthen J (1976). Properties of the K562 cell line, derived from a patient with chronic myeloid leukemia. Int J Cancer.

[pgen-0030136-b014] The_ENCODE_Consortium (2007). Identification and analysis of functional elements in 1% of the human genome by the ENCODE pilot project. Nature.

[pgen-0030136-b015] Kim TH, Barrera LO, Zheng M, Qu C, Singer MA (2005). A high-resolution map of active promoters in the human genome. Nature.

[pgen-0030136-b016] Kim TH, Abdullaev ZK, Smith AD, Ching KA, Loukinov DI (2007). Analysis of the vertebrate insulator protein CTCF-binding sites in the human genome. Cell.

[pgen-0030136-b017] Koch CM, Andrews RM, Flicek P, Dillon SC, Karaoz U (2007). The landscape of histone modifications across 1% of the human genome in five human cell lines. Genome Res.

[pgen-0030136-b018] Bailey TL, Elkan C (1994). Fitting a mixture model by expectation maximization to discover motifs in biopolymers. Proc Int Conf Intell Syst Mol Biol.

[pgen-0030136-b019] Wingender E, Dietze P, Karas H, Knuppel R (1996). TRANSFAC: A database on transcription factors and their DNA binding sites. Nucleic Acids Res.

[pgen-0030136-b020] Sandelin A, Alkema W, Engstrom P, Wasserman WW, Lenhard B (2004). JASPAR: An open-access database for eukaryotic transcription factor binding profiles. Nucleic Acids Res.

[pgen-0030136-b021] Bell AC, Felsenfeld G (2000). Methylation of a CTCF-dependent boundary controls imprinted expression of the Igf2 gene. Nature.

[pgen-0030136-b022] Bailey TL, Gribskov M (1998). Combining evidence using *p*-values: Application to sequence homology searches. Bioinformatics.

[pgen-0030136-b023] Siepel A, Bejerano G, Pedersen JS, Hinrichs AS, Hou M (2005). Evolutionarily conserved elements in vertebrate, insect, worm, and yeast genomes. Genome Res.

[pgen-0030136-b024] Frith MC, Fu Y, Yu L, Chen JF, Hansen U (2004). Detection of functional DNA motifs via statistical over-representation. Nucleic Acids Res.

[pgen-0030136-b025] Smith AD, Sumazin P, Xuan Z, Zhang MQ (2006). DNA motifs in human and mouse proximal promoters predict tissue-specific expression. Proc Natl Acad Sci U S A.

[pgen-0030136-b026] Wadman IA, Osada H, Grutz GG, Agulnick AD, Westphal H (1997). The LIM-only protein Lmo2 is a bridging molecule assembling an erythroid, DNA-binding complex which includes the TAL1, E47, GATA-1 and Ldb1/NLI proteins. EMBO J.

[pgen-0030136-b027] Robb L, Begley CG (1997). The SCL/TAL1 gene: Roles in normal and malignant haematopoiesis. Bioessays.

[pgen-0030136-b028] Pevny L, Simon MC, Robertson E, Klein WH, Tsai SF (1991). Erythroid differentiation in chimaeric mice blocked by a targeted mutation in the gene for transcription factor GATA-1. Nature.

[pgen-0030136-b029] Pesce M, Gross MK, Scholer HR (1998). In line with our ancestors: Oct–4 and the mammalian germ. Bioessays.

[pgen-0030136-b030] Chimal-Monroy J, Rodriguez-Leon J, Montero JA, Ganan Y, Macias D (2003). Analysis of the molecular cascade responsible for mesodermal limb chondrogenesis: Sox genes and BMP signaling. Dev Biol.

[pgen-0030136-b031] Raz R, Lee CK, Cannizzaro LA, d'Eustachio P, Levy DE (1999). Essential role of STAT3 for embryonic stem cell pluripotency. Proc Natl Acad Sci U S A.

[pgen-0030136-b032] Rosl F, Das BC, Lengert M, Geletneky K, zur Hausen H (1997). Antioxidant-induced changes of the AP-1 transcription complex are paralleled by a selective suppression of human papillomavirus transcription. J Virol.

[pgen-0030136-b033] Halazonetis TD, Georgopoulos K, Greenberg ME, Leder P (1988). c-Jun dimerizes with itself and with c-Fos, forming complexes of different DNA binding affinities. Cell.

[pgen-0030136-b034] Goodman RH, Smolik S (2000). CBP/p300 in cell growth, transformation, and development. Genes Dev.

[pgen-0030136-b035] Dostie J, Richmond TA, Arnaout RA, Selzer RR, Lee WL (2006). Chromosome Conformation Capture Carbon Copy (5C): A massively parallel solution for mapping interactions between genomic elements. Genome Res.

[pgen-0030136-b036] Crawford GE, Holt IE, Whittle J, Webb BD, Tai D (2006). Genome-wide mapping of DNase hypersensitive sites using massively parallel signature sequencing (MPSS). Genome Res.

[pgen-0030136-b037] Thomson JA, Itskovitz-Eldor J, Shapiro SS, Waknitz MA, Swiergiel JJ (1998). Embryonic stem cell lines derived from human blastocysts. Science.

[pgen-0030136-b038] Irizarry RA, Bolstad BM, Collin F, Cope LM, Hobbs B (2003). Summaries of Affymetrix GeneChip probe level data. Nucleic Acids Res.

[pgen-0030136-b039] Tuan DY, Solomon WB, London IM, Lee DP (1989). An erythroid-specific, developmental-stage-independent enhancer far upstream of the human “beta-like globin” genes. Proc Natl Acad Sci U S A.

[pgen-0030136-b040] Chung JH, Bell AC, Felsenfeld G (1997). Characterization of the chicken beta-globin insulator. Proc Natl Acad Sci U S A.

[pgen-0030136-b041] Ward JH (1963). Hierarchical grouping to optimize an objective function. J Am Stat Assoc.

