# Supplementary material for: Identification and Characterization of Cell Type–Specific and Ubiquitous Chromatin Regulatory Structures in the Human Genome
Source: PLoS Genet. 2007 Aug 17;3(8):e136. doi: 10.1371/journal.pgen.0030136 (PMC1950163; doi:10.1371/journal.pgen.0030136)
Supplement: Supplementary file 1 [file pgen.0030136.st001.doc]

**Supplementary table 1. The 26 ubiquitous distal DHS that overlap CTCF ChIP-chip hits:**

| ENCODE ID | Chromosome | Start Coordinate | End Coordinate | Nearby Genes |
| --- | --- | --- | --- | --- |
| **ENm002** | **chr5** | **131371030** | **131372094** | **inside first intron of ACSL6** |
| **ENm002** | **chr5** | **131428296** | **131429246** | **between IL3 and CSF2** |
| **ENm002** | **chr5** | **131788498** | **131791196** | **between SLC22A5 and IRF1** |
| **ENm002** | **chr5** | **131864346** | **131865600** | **between IRF1 and IL5** |
| **ENm002** | **chr5** | **131889844** | **131891212** | **between IRF1 and IL5** |
| ENm003 | chr11 | 116108008 | 116109528 | at the 3' of APOA5 |
| ENm004 | chr22 | 30697862 | 30698698 | between YWHAH and SLC5A1 |
| ENm004 | chr22 | 30353810 | 30355026 | inside first intron of PISD |
| ENm005 | chr21 | 33493445 | 33494129 | between OLIG1 and INFAR2 |
| ENm005 | chr21 | 34217763 | 34219283 | between ATP5O and MRPS6 |
| **ENm011** | **chr11** | **2014898** | **2015506** | **between H19 and IGF2** |
| **ENm011** | **chr11** | **2129164** | **2130152** | **between IGF2 and INS/TH** |
| **ENm011** | **chr11** | **2175106** | **2175562** | **between INS/TH and ASCL2** |
| ENr111 | chr13 | 29604786 | 29605660 | between KATNAL1 and UBL3 |
| ENr212 | chr5 | 142072469 | 142073457 | between FGF1 and ARHGAP26 |
| ENr223 | chr6 | 73896277 | 73896771 | inside a 3' intron of KCNQ5 |
| ENr223 | chr6 | 74154829 | 74155969 | between KCNQ5 and DDX43 |
| ENr231 | chr1 | 148379900 | 148380584 | between TUFT1 and SNX27 |
| ENr232 | chr9 | 128769682 | 128770176 | inside second intron of PHYHD1 |
| ENr232 | chr9 | 128973096 | 128973932 | inside an intron of PPP2R4 |
| ENr233 | chr15 | 41768343 | 41770015 | in the first intron of KIAA |
| ENr322 | chr14 | 98802542 | 98802960 | inside first intron of BCL11B |
| ENr331 | chr2 | 220331877 | 220333055 | at the 3'end of SLC4A3 |
| ENr332 | chr11 | 64411785 | 64413001 | between EHD1 and KIAA0404 |
| ENr332 | chr11 | 64211905 | 64213197 | inside an intron of NRXN2 |
| ENr334 | chr6 | 41444731 | 41445187 | between NCR2 and FOXP4 |
