# Supplementary material for: Identification and Characterization of Cell Type–Specific and Ubiquitous Chromatin Regulatory Structures in the Human Genome
Source: PLoS Genet. 2007 Aug 17;3(8):e136. doi: 10.1371/journal.pgen.0030136 (PMC1950163; doi:10.1371/journal.pgen.0030136)
Supplement: Supplementary file 2 [file pgen.0030136.st002.doc]

# Supplementary table 2. Contingency tables of DHS vs. CTCF hits

A. All DHS in IMR90 cells

|  | **Overlap with**  **CTCF hits** | **Non-overlap with CTCF hits** |
| --- | --- | --- |
| **With CTCF motif** | **88** | **139** |
| **Without CTCF motif** | **72** | **945** |

B. Ubiquitous distal DHS

|  | **Overlap with**  **CTCF hits** | **Non-overlap with CTCF hits** |
| --- | --- | --- |
| **With CTCF motif** | **19** | **4** |
| **Without CTCF motif** | **7** | **7** |
