# Supplementary material for: Identification and Characterization of Cell Type–Specific and Ubiquitous Chromatin Regulatory Structures in the Human Genome
Source: PLoS Genet. 2007 Aug 17;3(8):e136. doi: 10.1371/journal.pgen.0030136 (PMC1950163; doi:10.1371/journal.pgen.0030136)
Supplement: Supplementary file 3 [file pgen.0030136.st003.doc]

**Supplementary Table 3. DNase HS sites used in enhancer blocking assay (hg17 coordinates)**

| **Clone** | **Chr** | **Start** | **End** | **Size** | **Unique Identifyer** | **Proximal or**  **Distal to TSS** |
| --- | --- | --- | --- | --- | --- | --- |
| **1** | **chr1** | **148126163** | **148127558** | **1396** | **Ins001** | **Proximal** |
| **2** | **chr11** | **2014455** | **2015684** | **1230** | **Ins002** | **Distal** |
| **3** | **chr2** | **220332056** | **220333640** | **1585** | **Ins023** | **Distal** |
| **4** | **chr5** | **131859695** | **131861263** | **1569** | **Ins034** | **Proximal** |
| **5** | **chr5** | **131864367** | **131865790** | **1424** | **Ins035** | **Distal** |
| **6** | **chr6** | **41678255** | **41679557** | **1303** | **Ins041** | **Proximal** |
| **7** | **chr6** | **41851127** | **41852406** | **1280** | **Ins044** | **Distal** |
