# Supplementary material for: Identification and Characterization of Cell Type–Specific and Ubiquitous Chromatin Regulatory Structures in the Human Genome
Source: PLoS Genet. 2007 Aug 17;3(8):e136. doi: 10.1371/journal.pgen.0030136 (PMC1950163; doi:10.1371/journal.pgen.0030136)
Supplement: Supplementary file 4 [file pgen.0030136.st004.doc]

Supplementary Table 4. Enrichment of AP-1 motif (TGASTCA) in DNase HS sites that overlap with P300 hits

|  | + AP-1 | - AP-1 | Occurrence% |
| --- | --- | --- | --- |
| Overlap with P300 hits | 85 | 129 | 40% |
| No overlap with P300 hits | 98 | 730 | 12% |
| Overlap% | 46% | 15% | P(χ^2^)<2.2e-16 |
